# Vibroarthrographic analysis of patellofemoral joint arthrokinematics during squats with increasing external loads

**DOI:** 10.1186/s13102-020-00201-z

**Published:** 2020-08-27

**Authors:** Ewelina Ołowiana, Noelle Selkow, Kevin Laudner, Daniel Puciato, Dawid Bączkowicz

**Affiliations:** 1 Faculty of Physical Education and Physiotherapy, Opole University of Technology, Prószkowska 76, PL-45-578 Opole, Poland; 2 Illinois State University, School of Kinesiology and Recreation, Normal, IL USA; 3 Beth El College of Nursing and Health Sciences, University of Colorado, Colorado Springs, CO USA

**Keywords:** Knee joint, Squat, Friction, Crepitus, Contact stress, Biomechanics

## Abstract

**Background:**

The patellofemoral joint (PFJ) provides extremely low kinetic friction, which results in optimal arthrokinematic motion quality. Previous research showed that these friction-reducing properties may be diminished due to the increase in articular contact forces. However, this phenomenon has not been analyzed in vivo during functional daily-living activities*.* The aim of this study was the vibroarthrographic assessment of changes in PFJ arthrokinematics during squats with variated loads.

**Methods:**

114 knees from 57 asymptomatic subjects (23 females and 34 males) whose ages ranged from 19 to 26 years were enrolled in this study. Participants were asked to perform 3 trials: 4 repetitions of bodyweight squats (L0), 4 repetitions of 10 kg barbell back loaded squats (L10), 4 repetitions of 20 kg barbell back loaded squats (L20). During the unloaded and loaded (L10, L20) squats, vibroarthrographic signals were collected using an accelerometer placed on the patella and were described by the following parameters: variation of mean square (VMS), mean range (R4), and power spectral density for frequency of 50–250 Hz (P1) and 250–450 Hz (P2).

**Results:**

Obtained results showed that the lowest values were noted in the unloaded condition and that the increased applied loads had a significant concomitant increase in all the aforementioned parameters bilaterally (*p* < 0.05).

**Conclusion:**

This phenomenon indicates that the application of increasing knee loads during squats corresponds to higher intensity of vibroacoustic emission, which might be related to higher contact stress and kinetic friction as well as diminished arthrokinematic motion quality.

## Background

The knee is one of the most loaded joints within the human organism which results in its considerable susceptibility to injuries and an increased risk of early degeneration of the articular surface [1, 2]. The patellofemoral joint (PFJ) plays a key role in the knee extensor mechanism since the patella, being the largest sesamoid bone, increases the length of the lever arm of the patellar tendon, which improves the quadriceps strength by 30–50% [3]. Additionally, the patella acts as a bony shield for the anterior trochlea and due to its interposed position in the extensor apparatus, protects against excessive friction between the quadriceps tendon and the femoral condyles [4]. An indication that the PFJ adapts to considerable loading is the thickness of the hyaline cartilage. It may reach 6–7 mm thick at the central part of patella, which constitutes the greatest thickness among synovial joints of the human musculoskeletal system [5, 6].

Physiological hyaline cartilage along with synovial fluid remarkably decreases the coefficient of kinetic friction, which determines efficiency of the knee extensor apparatus and helps protect against high compressive loads [7]. Using computer models or cadaveric specimens, researchers have shown that smooth and lubricated articular surfaces provide an extremely low level of friction, which fluctuates around 0.002 μ and 0.02 μ, depending on testing conditions [8, 9]. Within in vivo conditions this phenomenon is clearly visible in the evaluation of joint motion quality via vibroarthrography (VAG), a method based on the analysis of micro vibrations generated during relative movement of articular surfaces [10, 11]. This research has shown that young and healthy joints provide optimal, smooth and practically vibration free arthrokinematics [12, 13]. Using VAG, it was found that the there are many confounders of friction related to the qualitative aspects of arthrokinematics, including age-related alterations and degenerative changes within synovial joint environment, which directly translate into quicker degeneration of joint surfaces [14, 15].

It has been previously shown that VAG possess not only high accuracy and specificity when differentiating synovial joints’ deteriorations with various biomechanical and morphological origins, but also is sensitive for identifying the changes in arthrokinematics related to the level of the joint load at the end of a performed task [16–20]. From an arthrology perspective, this finding seems to be particularly important, because the load on articular surfaces is one of the most essential factors affecting the level of kinetic friction and joint wear [7, 21]. Meanwhile, most of the previously presented research associated with analysis of load impact on articular friction and contact stress has been based on mathematical modelling or ex vivo analysis, which constitutes a considerable limitation in their clinical application [22–28]. Thus, it seems that a noninvasive method, such as vibroarthrography, might provide a new perspective on PFJ arthrokinematics analyzed in vivo. Furthermore, as it was previously postulated, the subsequent VAG analyses should consider the influence of the load level on knee arthrokinematics during functional closed kinetic chain activities [19]. In comparison to analyses of open chain activities, the closed kinetic chain provides a better reproduction of the character of the PFJ in daily activities [23, 29, 30].

Hence, the objective of this study was to use vibroarthrographically assess the impact of load on PFJ arthrokinematics, analyzed in vivo during the squat motion. It was hypothesized that the level of vibroacoustic emission would be considerably higher during squats with increasing loads when compared to unloaded squats. We assume that the above-mentioned load-associated increase of vibroacoustic emission might be largely driven by the increase of kinetic friction and contact stress within the PFJ. The results of this study might aid clinicians’ understanding of the relationship between the level of articular surfaces compressive loads and qualitative aspects of arthrokinematics considered as friction-reducing properties of diarthrosis. Furthermore, these results could broaden knowledge related to the joint biotribology, and might benefit clinicians, because the squat has long been a basic element of strength training among athletes, as well as is various rehabilitative protocols [31–34]. Because PFJ disorders are commonly encountered in sports and rehabilitation, better recognition of biomechanical behavior of the PFJ during loaded and unloaded movements is essential in order to treat it effectively [4].

## Methods

### Participants

A convenience sample of asymptomatic volunteers was recruited from students of the Faculty of Physical Education and Physiotherapy of Opole University of Technology, Poland. To be included in this study, the participants were expected to be able to easily perform a series of squats with external loads of 10 kg and 20 kg. The external load of 10 kg constituted approximately 13% of body weight (BW) for men and 18% for women, whereas the percentage values for men and women doubled respectively for the external load of 20 kg. Additionally, the inclusion criteria included only participants with moderate physical activity level, according to the International Physical Activity Questionnaire (long form) [35, 36]. Moreover, only individuals with no history of knee disorder or other diagnosed injury or pathology within the lower extremity were included in the study group. The clinical evaluation of the analyzed group was based on both anamnesis (participant’s self-reported medical history) and physical examination performed by a senior physiotherapist, but without radiological exclusion of the cartilage pathologies. Finally, 114 healthy knee joints from 57 volunteers (23 females and 34 males) aged 19 to 26 years were enrolled in the study. Age, gender and anthropometric data in the analyzed volunteers are given in Table 1.

**Table 1:** Subject demographics (mean ± standard deviation)

	All group (*n* = 57)	Men (*n* = 34)	Women (*n* = 23)
Age (years)	20.7 ± 1.9	20.8 ± 2.3	20.6 ± 1.2
Height (cm)	174.3 ± 11.1	180.9 ± 8.1	164.4 ± 6.8*
Weight (kg)	68.7 ± 13.9	77.1 ± 11.3	56.0 ± 5.3*
BMI (kg/m^2^)	23.6 ± 3.0	23.5 ± 2.8	20.8 ± 1.8

*BMI* body mass index; *, statistically significant differences between men and women, *p* < 0.05

### Assessment of arthrokinematic motion quality

Based on previous studies, assessment of the PFJ arthrokinematic motion quality was performed with an accelerometer sensor placed 1 cm above the apex of the patella [11, 20, 37].

**Fig. 1:**
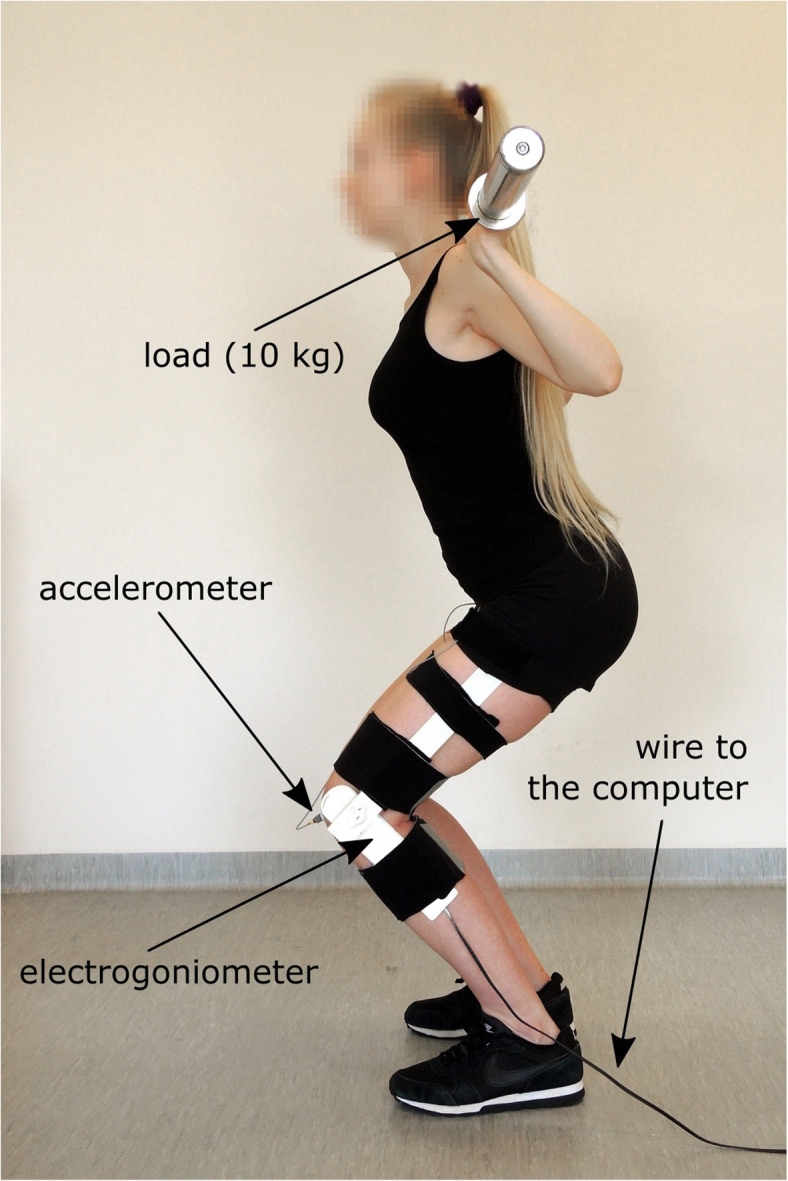
Participant during assessment (10 kg load)

This measurement was performed during both unloaded and loaded squat movements. In total, participants were asked to perform 3 trials: (i) 4 repetitions of bodyweight squats (L0); (ii) 4 repetitions of 10 kg barbell back loaded squats (L10); (iii) 4 repetitions of 20 kg barbell back loaded squats (L20), with one-minute rest between each test. The order of the trials was randomly selected. In the unloaded trials, the squats were performed with the hands placed behind the head, while loaded trials used barbells of 10 kg and 20 kg (Fig. 1). In all these conditions, subjects were instructed to execute the squat from a neutral position (approximately 0° of knee flexion) to the depth of approximately 90° of knee flexion while maintaining heel contact with the floor. The constant velocities of flexion/extension movements were kept at 48 beats per minute with a metronome and the angle of the knee joint was measured using an electrogoniometer. Each squat test lasted 10 s, during which four cycles of squats were performed. Before data collection, each subject performed the squat maneuver within the protocol guidelines. Verbal feedback regarding both the depth and duration of the movement was provided after each training squat. The trials were repeated if the requirements were not fulfilled. An average of 4 attempts were required to obtain the 3 acceptable trials.

The VAG signals were collected using an acceleration sensor, Brüel & Kjær model 4513B-002, with a multi-channel Nexus conditioning amplifier (Brüel & Kjær Sound & Vibration Measurement A/S, Denmark). Data were recorded at a sampling frequency of 10 kHz and then filtered using a fourth-order zero-phase Butterworth band-pass digital filter with cutoff frequencies between 50 Hz and 1000 Hz. The variability of the VAG signal in the time domain was assessed by computing the following parameters [37, 38]:

1. the mean-squared values of an obtained signal in fixed-duration segments of 5 ms each and then computing the variance of the values of the parameter over the entire duration of the signal (VMS) [39];
2. signal amplitude was calculated as the difference between the mean of the four most prominent peaks and the mean of the four most prominent troughs of the VAG signal (R4).

The frequency characteristics of the VAG signal were examined by a short-time Fourier transform analysis. The short-time spectra were obtained by computing the discrete Fourier transform of segments, 150 samples each, Hanning window, and 100 samples overlap of each segment. The spectral activity was analyzed by summing spectral power of the VAG signal in two bands: 50–250 Hz (P1) and 250–450 Hz (P2) [18, 37–40].

### Statistical analysis

Normality of the distribution was assessed with the Shapiro-Wilk test. Because of a skewed distribution of VMS, R4, P1, and P2 parameter values, they were analyzed using a logarithmic transformation. Statistical significance of changes between assessment conditions (load level 0, 10 and 20 kg) was performed with one-way analysis of variance (ANOVA) with repeated measures, and then the Tukey’s honest significant difference (HSD) test for post-hoc comparisons. Differences in values of VAG parameters between left and right lower limbs were evaluated by dependent t-test for paired samples. For examination of correlations between analyzed variables, Pearson r tests were performed. The level of significance was set at *p* < 0.05. Statistical analyses were performed using Statistica version 10 (TIBCO Software Inc., Palo Alto, CA).

## Results

**Fig. 2:**
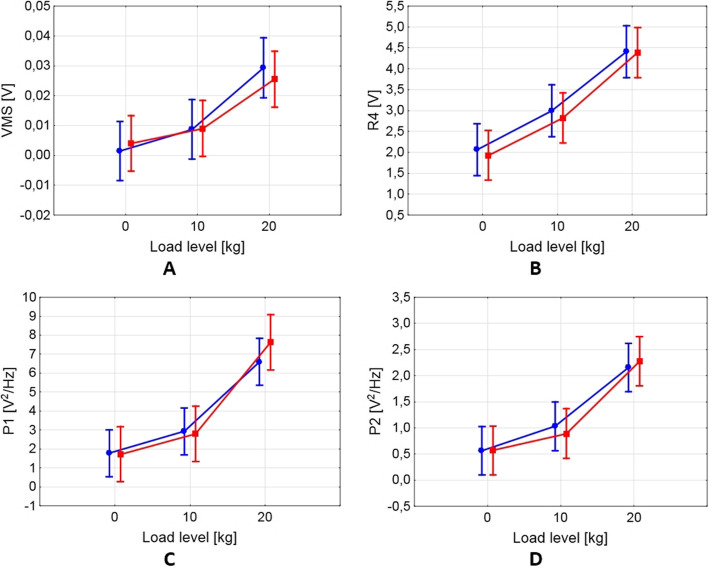
Values of vibroarthrographic parameters in the conditions analyzed (blue round marks, left limb; red square marks, right limb)

**Fig. 3:**
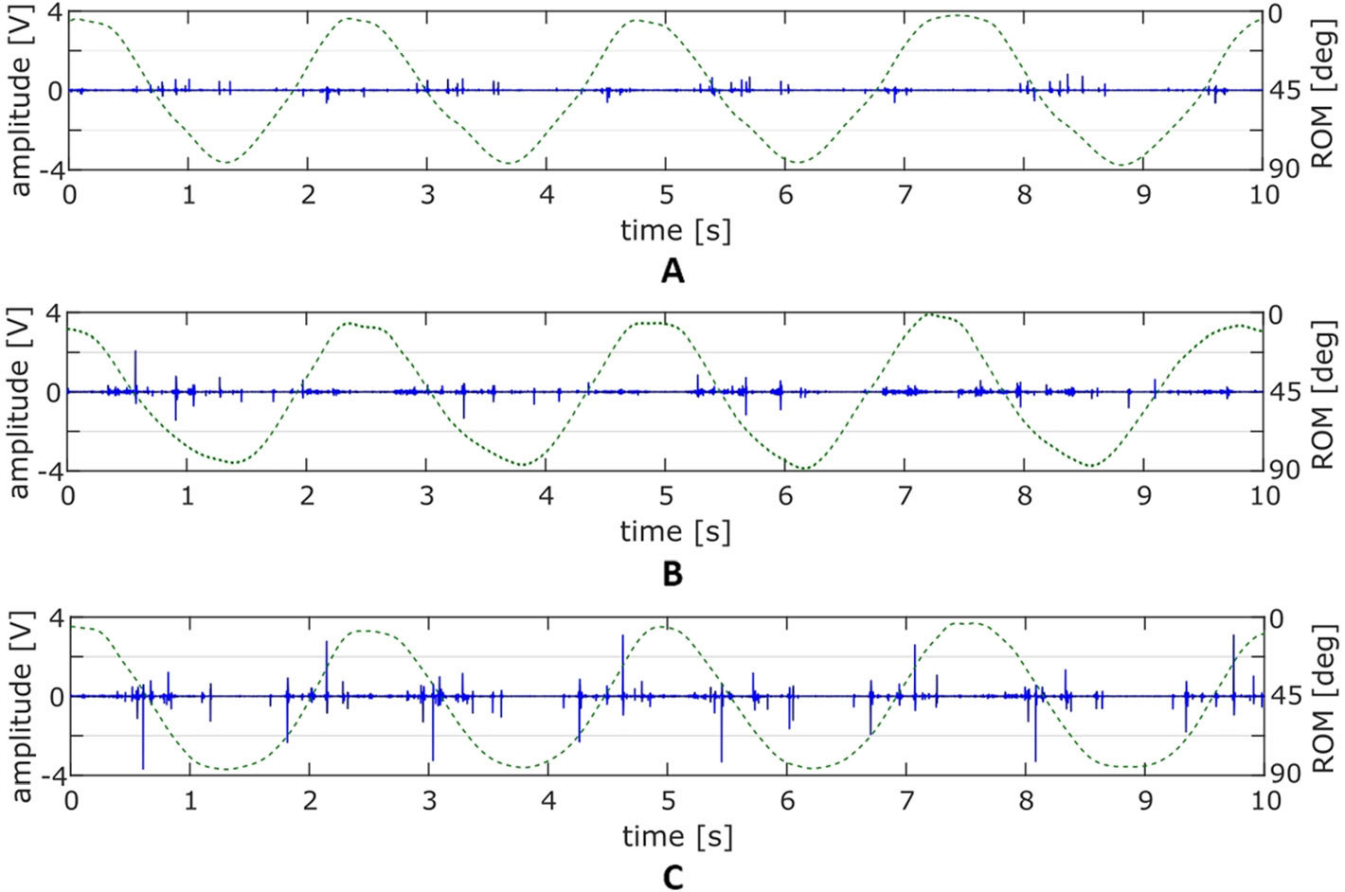
The course of representative vibroarthrographic signals for conditions (**a**) with no load (**b**) with 10 kg load (**c**) with 20 kg load

Calculated values of analyzed VAG parameters (VMS, R4, P1 and P2), expressed as median and variance values are presented in Fig. 2 and Table 2. Moreover, for an additional expression of the VAG signals characteristics, representative plots of the vibroarthrographic registered time-series, specific for each load-related condition (L0, L10 and L20) are shown in Fig. 3. The plots contain acquired signal course expressed in volts (blue solid line, left y scale) and registered position of the joint (ROM) via electrogoniometer expressed in degrees (green dotted line, right y scale).

**Table 2:** Parameters of vibroarthrographic signals under different loads

	VMS [V] median ± variance		R4 [V] median ± variance		P1 [V^2^/Hz] median ± variance		P2 [V^2^/Hz] median ± variance	
	Left	Right	Left	Right	Left	Right	Left	Right
No load (L0)	0.001 ± 0.004	0.004 ± 0.019	2.06 ± 1.24	1.93 ± 1.62	1.77 ± 1.51	1.72 ± 2.49	0.57 ± 0.88	0.57 ± 1.08
10 kg load (L10)	0.009 ± 0.021	0.009 ± 0.026	3.00 ± 1.92	2.82 ± 2.00	2.92 ± 2.62	2.80 ± 3.92	1,03 ± 1,24	0.89 ± 1.24
20 kg load (L20)	0.029 ± 0.060	0.026 ± 0.051	4.41 ± 3.27	4.38 ± 2.85	6.60 ± 7.37	7.63 ± 8.10	2.16 ± 2.56	2.28 ± 254
*p*-values								
L0 vs L10	**0.019**	**0.005**	**0.008**	**0.003**	**0.027**	**0.031**	**0.006**	**0.007**
L10 vs L20	0.102	**0.019**	**0.044**	**0.032**	**0.006**	**0.002**	**0.031**	**0.009**
L0 vs L20	**< 0.001**	**< 0.001**	**< 0.001**	**< 0.001**	**< 0.001**	**< 0.001**	**< 0.001**	**< 0.001**

*VMS* variability of the mean squares calculated in 5 ms windows; R4, mean of four maximal and four minimal values; P1, P2, power spectral density bands: 50–250 Hz and 250–450 Hz, respectively; bolded values indicate statistically significant differences

The performed ANOVA revealed that there was a significant main effect of the applied load on values of each analyzed parameter, similar in both lower limbs. For the left limb the results were as follows: F = 11.69, *p* < 0.001; F = 15.30, *p* < 0.001; F = 16.70, *p* < 0.001; F = 16.64; *p* < 0.001, for VMS, R4, P1 and P2, respectively, and analogously for the right limb: F = 17.94, *p* < 0.001; F = 17.75, *p* < 0.001; F = 18.35; *p* < 0.001; F = 19.02. *p* < 0.001. Subsequently, using the post-hoc test we identified that the rising level of the applied load has a significant impact on increase of all VAG parameters, in both lower limbs (Fig. 2 and Table 2). The lowest values of VMS, R4, P1 and P2 parameters were noted during motion without additional load (L0). These values reflect the characteristics of the signals course typical for this condition, when signals possessed low amplitude and variability, only with small, occasional peaks (Fig. 3a). Application of 10 kg load (L10) resulted in a statistically significant increase of all parameters (Table 2). It is also seen in Fig. 3b is that the motion with additional load generated signals with higher amplitude and variability, as compared to the L0 assessment. However, the highest values of VMS, R4, P1 and P2 parameters were observed in L20 condition, which were significantly higher than in the L10 condition, except comparison of the left limb VMS (Fig. 2 and Table 2). In this situation, when movements were performed under 20 kg load, the VAG signals possessed a more complex course with high peaks, repeatable in each cycle of motion (Fig. 3c). It should be also noted, that when compared bilaterally, there were no VAG differences, in each load condition.

In our study we also analyzed the relationships between values obtained in the left and right lower limbs (Table 3). Statistical analysis showed the presence of moderate positive correlations, especially in the P1 parameter, for which interactions were significant in all load-related conditions. Nevertheless, for VMS, R4 and P2 correlations have been also established, but only in the L10 and L20 assessments. Moreover, there were some dependencies in all VAG values when L0 was compared with L10, and L10 with L20 trials (Table 4).

**Table 3:** Values of correlation coefficient between parameters of vibroarthrographic signals recorded for left and right lower limbs

		VMS	R4	P1	P2
No load	R	0.16	0.13	**0.28**	0.14
	*p*	0.245	0.349	0.041	0.297
10 kg load	R	**0.44**	**0.47**	**0.42**	**0.31**
	*p*	0.001	0.000	0.002	0.022
20 kg load	R	**0.35**	**0.29**	**0.41**	**0.36**
	*p*	0.011	0.032	0.002	0.008

R, Spearman’s rank correlation coefficient; *p*, value of statistical significance; VMS, variability of the mean squares calculated in 5 ms windows; R4, mean of four maximal and four minimal values; P1, P2, power spectral density bands: 50–250 Hz and 250–450 Hz; bolded values indicate statistically significant differences

**Table 4:** Values of correlation coefficient between parameters of vibroarthrographic signals in different load-related conditions

		VMS	R4	P1	P2
No load &10 kg	R	**0.29**	**0.32**	**0.26**	**0.31**
	*p*	0.004	0.001	0.007	0.001
10 kg & 20 kg	R	**0.24**	**0.29**	**0.30**	**0.30**
	*p*	0.014	0.003	0.003	0.003
No load & 20 kg	R	0.07	0.05	0.02	0.04
	*p*	0.477	0.625	0.879	0.659

R, Spearman’s rank correlation coefficient; *p*, value of statistical significance level; VMS, variability of the mean squares calculated in 5 ms windows; R4, mean of four maximal and four minimal values; P1, P2, power spectral density bands: 50–250 Hz and 250–450 Hz. ^\^Bolded values indicate p < 0.05; bolded values indicate statistically significant differences

## Discussion

Following previous publications, our experiment assumed that the intensity of acquired vibroacoustic emission waves is closely associated with the kinetic friction and represents friction-induced vibrations generated by relative sliding and rolling of adjacent articular surfaces. From biomechanical and tribological points of view the ability of the articular surfaces to move smoothly against each other with low frictional noise indicates optimal arthrokinematic motion related with low kinetic friction, and as a result contributes to slow wear of cartilage [20]. The presented findings of this study confirm that during a knee flexion-extension movement with the load of the body weight, the PFJ arthrokinematic motion possesses low-vibration characteristics, despite relatively high values of articular surface load, typical for this analyzed condition. It has been experimentally proven via cadaveric and mathematical models that the contact forces of the PFJ articular surfaces while performing a squat may even reach 3–3.5 kN and the peak contact pressure equals ~ 8–9 MPa at 90° of knee flexion, which corresponds to multifold values of human body weight [30, 31, 41]. However, due to the friction-reducing properties of synovial joints, the coefficient of friction, which is defined as the ratio between strength required to generate gliding motility and pressure force of the body towards a particular surface, is still very low in the mentioned condition. It fluctuates around 0.03 μ, which is only slightly higher than coefficient of friction values typical for unloaded PFJ motion [42–44]. In our study this phenomenon is observed as relatively smooth and flat VAG signals, representing low vibroacoustic emission. Both variability (VMS) and amplitude (R4) of representative signals are characterized by low values. Especially the low values of the R4 parameter that indicate a lack of repetitive large peaks, whose presence often corresponds with macroscopic joint cartilage abnormalities or significant maltracking of articular surfaces [18, 38]. In turn, low spectral activity analyzed by summing spectral power of the VAG signal in two bands: 50–250 Hz (P1) and 250–450 Hz (P2) seems to be related with intact integrity of the superficial layer of hyaline cartilage and an efficient lubrication mechanism [37, 45].

Nonetheless, in the present study we predominantly focused on the impact of the external load on the quality of PFJ arthrokinematic motion. As expected, the PFJ frictional noise raised near-linearly with increasing levels of external load. The application of 10 kg external load resulted in an average 3-fold higher variability of recorded signal (VMS) and an average ~ 1.5-fold increase of amplitude and spectral activity (R4, P1 and P2). Furthermore, the squats performed with the 20 kg load were characterized by further, analogous increase of the VAG signal variability/amplitude and higher, 2.5-fold increase of spectral power in band 50–450 Hz, when compared with the 10 kg load. Similar results have been presented by Andersen et al. [19], who also found divergences between different loads for all VAG parameters. However, this previous study was based on an open kinetic chain analysis and the applied loads (up to 5 kg) were lower than the loads analyzed in the current study, which are more typical for different activities of daily living and sports. Moreover, although the absolute values between studies differed as a result of disparate parameters’ settings being used, similar tendencies have been observed.

Research has previously reported that squatting with an external load results in significantly greater patellofemoral joint reaction force and stress compared to an unloaded squat, and that the increase is proportional to the applied load [41]. Authors showed that a squat performed under a 35% of body weight external load yielded a 44% increase in PFJ stress across all knee flexion angles, with a peak patellofemoral contact stress of 13.06 MPa at 90° of the knee flexion, whereas when unloaded, the peak patellofemoral joint contact stress equaled 9.06 MPa. Such elevated values of contact stress might cause very high frictional shear stress during gliding motion of the patella, which consequently dramatically increases kinetic friction and related frictional noise [44]. It should be also noted that the observed increase of VAG signal parameters might be the result of load-related decline in lubrication efficiency. Although many different lubrication mechanisms have been proposed, it is becoming increasingly accepted that mainly a fluid film lubrication is responsible for the low friction in synovial joints, especially at high loads [46, 47]. Fluid film lubrication involves a thin synovia film that provides separation of the joint surfaces. Synovia being squeezed out of cartilage into the joint space as loading increases creates a thicker layer of film. Therefore, a decreased coefficient of friction with increased contact stress has been reported in many studies [48]. This observation may explain why the results reported by Ladly et al. [49] indicated that a 3 pound (1.4 kg) external loading of the patellofemoral joint has an insignificant effect on VAG signal power. Nonetheless, it is generally accepted that when the contact stress reaches a medium level (4–8 MPa), the velocity of fluid exuded from the cartilage reaches its limit and there is a concomitant increase in the coefficient of friction (frictional noise) thereby, increasing contact stresses [44].

Since all participants used in this study were subjected to the same level of load, we were also able to assess intra-group relationships between the changes in characteristics of VAG signal courses under the influence of applied load and the anthropological data of the analyzed cohort. We expected some dependencies, especially between the body weight and the VAG signal values, because contact stress (considered as the main determinant of observed changes) is a parameter that reflects distribution of load and strongly depends on the area of contact between articular surfaces. However, there were no statistically significant correlation between body weight and values of VAG signal parameters. Nevertheless, weak but statistically significant correlations were noted for all the considered parameters for trials L0 and L10 as well as trials L10 and L20. This shows that subjects who had a low level of vibroacoustic emission during the bodyweight squats also obtained a low level during the loaded conditions. Thereafter, we discovered weak and moderate positive dependencies in values of all VAG parameters between the left and right lower limbs, but only for the loaded trials. Hence, there is an assumption that occurrence of the mentioned dependencies might indicate the presence of ontogenetic conditionings, associated with the friction-reducing properties of synovial joint environment and different levels of load tolerance. It is, however, a complex phenomenon that requires further extensive research.

In the light of findings reported in the current investigation, there are several limitations that should be noted. Consideration should be given that the conducted analyses were limited to asymptomatic young and agile subjects, who were performed squats with different relative external loads. So, although the present study provides crucial insights into the PFJ function during squatting with and without loads, caution should be exercised in extrapolating these data to older patient populations or those suffering from anterior knee pain syndrome. Moreover, the inclusion/exclusion criteria were based on a medical interview, but not confirmed by advanced imaging procedures. Therefore, we cannot exclude that knees of some subjects revealed minor asymptomatic cartilage changes which could have affected our results. Another potential limitation is the fact that the VAG is a method of indirect arthrokinematic analysis focused on acquisition of the vibroacoustic signal using single skin-mounted accelerometer. Therefore, it should be clearly emphasized that the presented method allows for observation of only the effect of the kinetic friction phenomenon in the form of frictional noise which holds a multicomponent character. Moreover, it should be considered, that the accelerometer (despite the application of optimal localization) might detect the vibrations not only from the PFJ but also from tibiofemoral joint or other tissues [50]. However, the mechanical properties of synovial joints related to the friction and wear cannot be directly measured in vivo. Thus, it is generally accepted that the VAG method, despite some limitations, might provide sufficient, clinically meaningful information about the function and biotribological changes in whole articular environment.

Accordingly, the presented results are clinically applicable, because weight-bearing exercises, such as the squat, are frequently used during rehabilitation and are specific to many activities of daily living and sport activities. The ability to understand how friction related aspects of PFJ arthrokinematics vary among weight-bearing exercises with different loads will allow clinicians and trainers to prescribe safer and more effective rehabilitation treatments for patients and athletes during training. This is a particularly significant issue because excessive joint friction has been linked to articular cartilage degradation and may contribute to premature wear and knee pathologies (e.g. chondromalacia or osteoarthritis).

## Conclusions

The study constitutes a significant insight into the behavior of knee joint under external load conditions, specific to many functional activities. The presented results have shown that the application of increasing knee loads during squats corresponds to higher intensity of vibroacoustic emission, which might be related to higher contact stress and kinetic friction. However, it should be emphasized that these results are valid for asymptomatic healthy and young subjects.

## Data Availability

The datasets used and/or analyzed during the current study are available from the corresponding author on reasonable request.

## Abbreviations

PFJ: Patellofemoral joint
VAG: Vibroarthrography
